# A Rare Case of Early Corneal Blood Staining After Post-operative Hyphema in a Child With Congenital Glaucoma and Haab’s Striae

**DOI:** 10.7759/cureus.97269

**Published:** 2025-11-19

**Authors:** Eman A Atallah, Sayed Mohsen Alalawi, Sara H Alhendi, Yomna Alhalai, Amgad El Nokrashy

**Affiliations:** 1 Department of Ophthalmology, Faculty of Medicine, Mansoura University, Mansoura, EGY

**Keywords:** corneal staining, glaucoma surgery complications, haab's striae, hyphema, infantile glaucoma, pediatric ophthalmology

## Abstract

Primary congenital glaucoma is a rare disease affecting children early in life that has long been a challenging and serious condition requiring immediate intervention to prevent visual handicap. This case report describes a child diagnosed with primary congenital glaucoma who developed corneal blood staining despite low intraocular pressure following circumferential suture trabeculotomy with the presence of breaks in the Descemet's membrane. The report explores the clinical presentation, surgical complications, and management strategies and provides insights into the complexities of treating congenital glaucoma with significant Haab’s stria.

## Introduction

Congenital glaucoma occurs before the age of three years, presenting as developmental glaucoma occurring due to inadequate drainage of aqueous humor caused by improper development of the trabecular meshwork (TM) and anterior chamber angle [1]. Congenital glaucoma has long been a challenging and serious condition requiring prompt intervention to prevent visual handicap. Haab's striae, a common finding in these infants, and hyphema, which is a common surgical complication, can further complicate the management [2,3]. In this case report, we discuss a child with infantile glaucoma who developed corneal staining early following glaucoma surgery and without high intraocular pressure (IOP), highlighting the diagnostic and therapeutic challenges involved.

## Case presentation

A four-month-old male infant presented with enlarging eyes, photophobia and lacrimation as described by the mother. There was no known family history of glaucoma or other ocular diseases. Examination under general anaesthesia was performed and a full ocular examination was attempted.

Ocular examination showed presence of bilateral large hazy cornea with a diameter of 13.5 mm in both eyes and Haab's striae more significant in the right eye. Intraocular pressure was measured with a handheld electronic tonometer (Tono-Pen) and was 32 mmHg in the right eye and 27 mmHg in the left eye. Pre-operative visual acuity assessment could not be performed due to severe corneal edema causing marked corneal cloudiness. In addition, the anterior chamber, lens and fundus couldn’t be examined clinically also due to corneal haze. Amplitude scan (A-scan) ultrasonography was performed; axial length was 23.4 mm in the right eye and 22.8 mm in the left eye. Brightness scan (B-scan) ultrasonography was performed and excluded any posterior segment pathology. The infant was diagnosed with bilateral primary congenital glaucoma.

The infant was managed with circumferential suture trabeculotomy in the right eye and conventional rigid probe trabeculotomy in the left eye one week apart. Transient postoperative hyphema was noted in both eyes and resolved spontaneously in the left eye. However, the infant presented with corneal blood staining in the right eye two weeks after surgery although the IOP was only 9 mmHg measured with Tono-Pen. Ultrasound biomicroscopy (UBM) excluded the presence of a cyclodialysis cleft, and only a trabeculotomy cleft was noted. Conservative management and follow-up were adopted as the IOP was not elevated. In addition, the hyphema was less than half of the anterior chamber. Therefore, the appearance of significant corneal staining was surprising.

**Figure 1:**
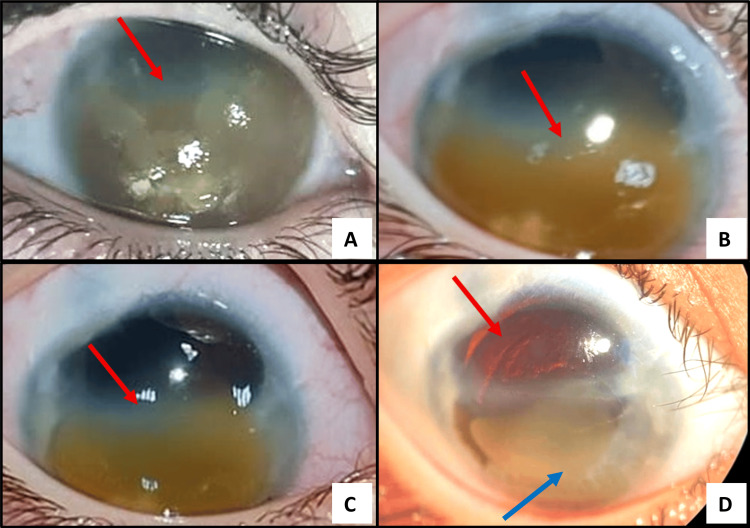
(A–D) Serial slit-lamp photographs of the right eye show the slow resolution of corneal blood staining over 20 months. (A) Two weeks post-operative, there is a dense reddish-brown stromal staining involving near total of the cornea (arrow). (B) At eight months post-operative, partial clearance is observed, progressing from the superior limbus downward (arrow). (C) At 14 months post-operative, further centripetal clearing is noted (arrow). (D) At 20 months post-operative, the superior third of the cornea appears clear (red arrow), while residual inferior opacity persists indicates stromal fibrosis, accompanied by peripheral iris atrophy and a fibrotic membrane bridging the inferior pupil of the right eye (blue arrow).

**Figure 2:**
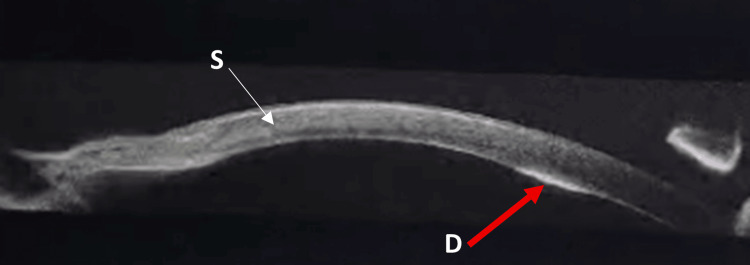
Anterior segment OCT result of the right cornea. Anterior segment OCT of the right cornea at 18-month follow-up showing a hyper-reflective thickened line (red arrow) indicating healed Descemet’s membrane breaks, and stromal heterogeneity suggestive of corneal remodeling due to persistent edema and previous insult. OCT: optical coherence tomography, S: Stromal layer, D: Descemet’s membrane.

The corneal blood staining showed a slow resolution over 20 months from the cornea. Peripheral corneal opacification, iris atrophy, retraction, lower membrane bridging the lower half of the pupil and finally complete acquired aniridia was noted (Figure 1). Anterior segment optical coherence tomography (OCT) at 18 months follow-up showed hyper-reflective areas at the level of Descemet’s membrane coinciding with Haab’s stria and opacified intervening stroma that can indicate migration of endothelial cells and laying of new basement membrane to fill the previous defect in endothelial cells or hyper-proliferation of pre-Descemet’s layer (Figure 2) [4].

Post-operatively, during visual assessment with the left eye covered, a searching movement was noticed, indicating amblyopia in the right eye. The mother was instructed to perform amblyopia therapy by patching the left eye; however, the infant became distressed and repeatedly removed the patch, suggesting deep amblyopia. Meanwhile, the slow clearance of the visual axis resulted in deep amblyopia in this eye. 

The option of early keratoplasty was discussed with the parents but their fear of possible complications kept them in follow-up option with its risk of amblyopia.

## Discussion

Primary congenital glaucoma is associated with several characteristic corneal changes, including corneal enlargement related to increased IOP, increased corneal thickness, corneal clouding and edema caused by Descemet’s membrane tears (Haab’s striae) [5]. Recurrent elevations in IOP can induce tears in Descemet’s membrane, with the ruptured edges retracting and curling inward to expose underlying stromal tissue [6]. Furthermore, corneal changes may also arise from surgical manipulation; during trabeculotomy, excessive anterior rotation of the instrument may lead to peripheral Descemet’s membrane detachment, although such detachments are generally minor and self-resolving [7]. Corneal decompensation has been reported in an adult patient with extensive Haab’s striae who had previously undergone treatment for congenital glaucoma, despite well-controlled IOP. This finding suggests that Haab’s striae may represent a form of endothelial vulnerability predisposing the cornea to later decompensation [8].

Corneal blood staining could be explained by deposition of extra- and intracellular hemoglobin particles as well as intracellular hemosiderin in the corneal stroma which can lead to opacification of the cornea [9]. Although, it typically occurs secondary to hyphema and elevated IOP. This condition is particularly concerning in children during the amblyogenic period, as the densely packed yellow-brown stromal pigmentation tends to clear slowly in a centripetal manner, beginning at the limbus and progressing centrally. Corneal endothelial dysfunction and the presence of Descemet’s membrane breaks are recognized as risk factors that predispose the cornea to blood staining [10]. Many authors reported that spontaneous resolution of corneal blood staining takes about two to three years [11,12]. Corneal blood staining most commonly occurs in the presence of elevated IOP; however, it may also develop despite normal or even low IOP levels. Beyer et al. reported early-onset corneal blood staining in two patients following ocular trauma, both of whom exhibited normal or only mildly elevated IOP [13].

Given the relatively high graft failure rate associated with pediatric keratoplasty and the challenges of visual rehabilitation in infants and young children, prevention of corneal blood staining remains paramount. Early recognition and timely intervention in eyes at risk are therefore essential to minimize irreversible corneal damage [5]. Adjunctive pharmacological options have been rarely used. Chan et al. mentioned that the use of oral deferiprone, a systemic iron chelator agent can facilitate the clearance of stromal hemoglobin deposits, leading to improved corneal transparency [14]. Nevertheless, it is not used in our case.

## Conclusions

Congenital glaucoma with significant Haab's striae and post-operative hyphema needs lower threshold for intervention and more frequent close follow-up examinations. Corneal blood staining is possible even without high IOP, especially in eyes with compromised corneal endothelium. Deep amblyopia is a concern in the young age group.
